# Competitive Growth of Ge Quantum Dots on a Si Micropillar with Pits for a Precisely Site-Controlled QDs/Microdisk System

**DOI:** 10.3390/nano13162323

**Published:** 2023-08-12

**Authors:** Jia Yan, Zhifang Zhang, Ningning Zhang, Qiang Huang, Yan Zhan, Zuimin Jiang, Zhenyang Zhong

**Affiliations:** 1 State Key Laboratory of Surface Physics, Department of Physics, Fudan University, Shanghai 200438, China; 2 Wide Bandgap Semiconductor Technology Disciplines State Key Laboratory, School of Microelectronics, Xidian University, Xi’an 710071, China

**Keywords:** site-controlled quantum dot, self-assembly, growth kinetics, QDs/microdisk system, spatial matching

## Abstract

Semiconductor quantum dots (QDs)/microdisks promise a unique system for comprehensive studies on cavity quantum electrodynamics and great potential for on-chip integrated light sources. Here, we report on a strategy for precisely site-controlled Ge QDs in SiGe microdisks via self-assembly growth of QDs on a micropillar with deterministic pits and subsequent etching. The competitive growth of QDs in pits and at the periphery of the micropillar is disclosed. By adjusting the growth temperature and Ge deposition, as well as the pit profiles, QDs can exclusively grow in pits that are exactly located at the field antinodes of the corresponding cavity mode of the microdisk. The inherent mechanism of the mandatory addressability of QDs is revealed in terms of growth kinetics based on the non-uniform surface chemical potential around the top of the micropillar with pits. Our results demonstrate a promising approach to scalable and deterministic QDs/microdisks with strong light–matter interaction desired for fundamental research and technological applications.

## 1. Introduction

The semiconductor quantum dot (QD) in a microdisk, i.e., QD/microdisk, has been of great interest for the comprehensive investigation of solid-state cavity quantum electrodynamics (CQED) [1,2,3], cavity spin dynamics [4], and quantum optics [5]. It is also a promising candidate for single-photon sources [6] and unique lasers [7,8,9] serving as on-chip-integrated light sources for next-generation chip-scale data communication networks and data centers [10], all-optical flip-flops for optical random-access memory [11], or label-free sensors (detectors) with ultrahigh sensitivity [12]. A key issue of QD/microdisks is to enhance the light–matter interaction by improving coupling between QDs and the cavity mode of the microdisk. It is well known that the general mode in the microdisk, whispering-gallery mode (WGM), is non-uniform in both the spectral and spatial domains [13]. In addition, the spectra of WGM are sensitive to the microdisk’s diameter and/or thickness [8,13], local curvature [14], small refractive index changes in the surrounding medium [15], temperature changes [7,16], surface adsorption of particles [17], and disruptions in the WGM evanescent field [12]. Such features provide various approaches to the spectral matching between QDs and the WGM of the microdisk, which emerge as the main subject in the design and fabrication of QD/microdisks. On the other hand, to realize strong coupling between QDs and microcavity mode, the location of the QD at the field antinode of microcavity mode, i.e., spatial matching, is also in high demand [7,8,16,18,19]. So far, the semiconductor QD/microcavity is always obtained via lithography on a film embedded with self-assembled QDs, whereas the self-assembled QDs are randomly distributed in-plane. Accordingly, most QDs are weakly coupled to the microcavity mode due to the spatial mismatching [7,18,19], leading to a small Purcell factor [16] and/or a distinguished background emission [8]. Additional post-growth techniques are indispensable to locate QDs in a cavity for spatial matching, such as scanning electron microscopy (SEM) [18] or atomic force microscopy (AFM) [20] for photonic crystals, scanning confocal photoluminescence microscopy for a pillar microcavity [16], or photoluminescence imaging for a circular Bragg grating [19]. At least two steps of locating QDs and the subsequent lithography render the fabrication procedures complex and result in more positional uncertainty. This is not suitable for on-chip integration. Particularly, the fabrication of microdisks with QDs in desired positions remains a great challenge, although the site-controlled QDs can be realized in simple pre-patterned substrates [21,22,23]. The preliminary results demonstrate that QDs can be selectively grown at ~D/2 (D: the lateral size of QD) from the microdisk’s edge [24,25]. Given that the light field maximum of WGM is located at ~λ/2n (λ: wavelength of WGM, n: effective refractive index) from the microdisk’s edge, the spatial matching between QDs and the WGM is still not optimal, since D/2 is considerably smaller than ~λ/2n. By introducing additional circular mesas on the top of the micropillar, the spatial matching in the radial direction between the QDs and WGM in a microdisk can be achieved. This results in an enhanced Purcell effect [26], whereas the spatial matching in the azimuth direction is still uncertain. A deterministic and scalable fabrication strategy for locating QDs in a microdisk with an accuracy of tens of nanometers is a compelling appeal for the strong coupling between QDs and WGM in a microdisk.

In this report, we demonstrate an approach to accurately site-controlled Ge QDs in SiGe microdisks via self-assembly growth of QDs on a SiGe/Si micropillar with pits and subsequent selective etching of partial Si in KOH solution. The positions of pits are designed at the field antinodes of cavity mode via the finite-difference time-domain (FDTD) method. By optimizing the growth temperature and the amount of Ge, as well as the pit profile, Ge QDs are successfully controlled to self-assemble only in pits, rather than at the periphery of the micropillar. Such competitive growth of QDs is interpreted in terms of the growth kinetics and the non-uniform surface chemical potential (SCP) around the top of the micropillar with pits. Our results provide a unique strategy to precisely control the QDs’ position in a microdisk for strong light–matter interaction in a deterministic fashion.

## 2. Materials and Methods

**Figure 1:**
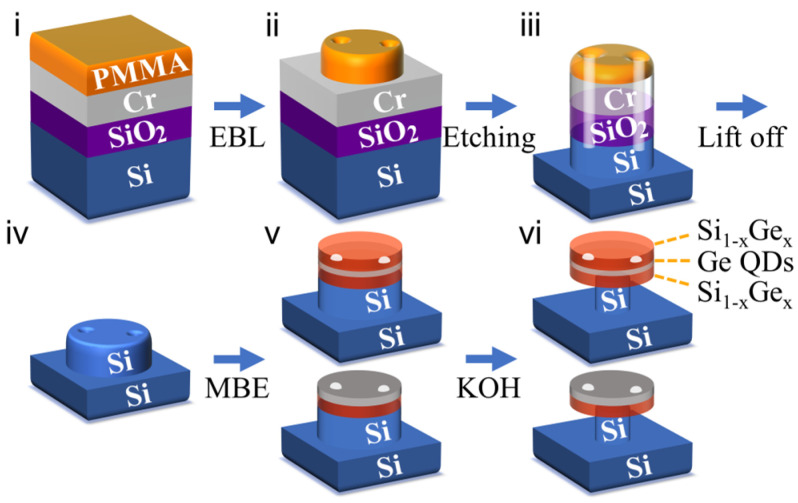
The schematic fabrication processes for the accurate site-controlling of Ge QDs in a SiGe microdisk. (**i**) Spin-coating of PMMA on a silicon substrate covered with thin films of SiO_2_ and Cr, (**ii**) initial pattern formation of PMMA, (**iii**) pattern transferring into the substrate by dry etching. The white cylinders represent the etched pits. (**iv**) a Si micropillar with deterministic pits after etching of SiO_2_ and Cr films, (**v**) the SiGe alloy layer and Ge QDs grown by MBE on the Si micropillar. The white dots indicate the site-controlled Ge QDs. (**vi**) a SiGe microdisk embedded with the site-controlled Ge QDs on a Si pedestal after the selective etching of Si in a KOH solution.

The fabrication process of precisely site-controlled Ge QDs in SiGe microdisks is schematically shown in Figure 1. In step (i), a SiO_2_ layer of 100 nm is grown on a Si (001) substrate by chemical vapor deposition, followed by a chromium (Cr) film of 20 nm by thermal evaporation. The PMMA pattern of the microdisk with pits can be defined by electron-beam lithography (EBL), as shown in step (ii). The pits are designed to be located at the field antinodes of the corresponding WGM of the final SiGe microdisk. After Ar^+^ ion-beam etching, the pattern is transferred into the hard mask of a Cr microdisk with pits. Further deep etching of Si is performed by inductively coupled plasma reactive ion etching of SF_6_ and C_4_F_8_, as demonstrated in step (iii). By dipping in HF solution, all masks on the silicon surface are removed. The silicon micropillar with pits on the top is obtained as shown in step (iv). It can be seen that smaller pits generally correspond to a smaller etching rate of Si in the pit. In the present case, the diameter and the height of the micropillar were 1.7 and 1.5 μm, respectively. The lateral size of the pits was in the range of 70–140 nm. The pre-patterned Si template was cleaned by the standard RCA method [27], followed by HF treatment to provide a H-terminated surface. After thermal desorption of the sample at 800 °C for 3 min, a Si buffer layer of 100 nm was grown at 430 °C by molecular beam epitaxy (MBE) in a Riber Eva-32 system. A Si_0.89_Ge_0.11_ alloy layer of 80 nm was subsequently deposited at 400 °C. The growth rates of Si and Ge are 0.58 Å·s^−1^ and 0.073 Å·s^−1^, respectively. Ge was then deposited to form self-assembled Ge QDs, as shown in step (v). Finally, a part of the Si micropillar under the SiGe alloy layer was selectively etched in a KOH solution to obtain a SiGe microdisk with Ge QDs supported by a silicon pedestal, as shown in step (vi). In order to fabricate the SiGe microdisk with embedded Ge QDs, an additional Si_0.89_Ge_0.11_ cap layer should be grown before the selective etching. The surface morphology without a cap layer was imaged by AFM (Veeco DI Multimode V SPM) in a tapping mode.

## 3. Results and Discussion

### 3.1. The Competitive Growths of QDs in Pits and at Periphery

**Figure 2:**
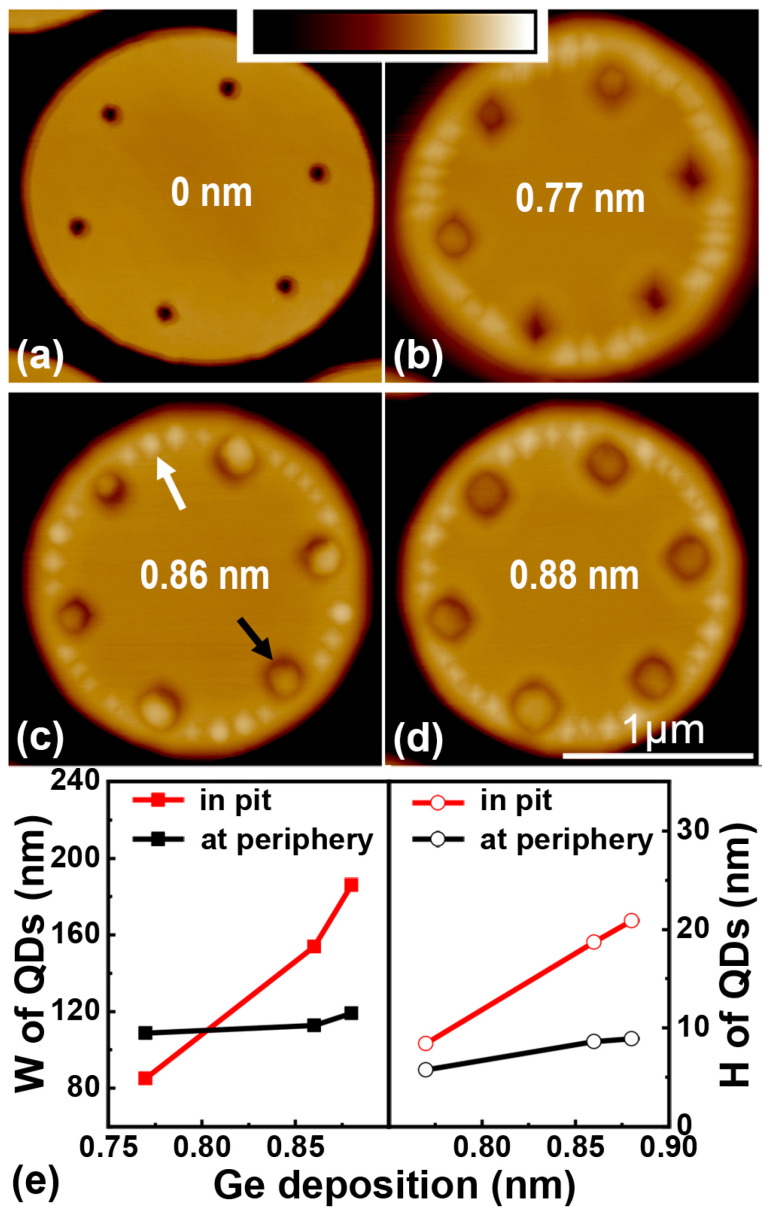
AFM images of QDs on the micropillars with pits for different Ge depositions. (**a**) The typical surface topography of a Si micropillar with deterministic pits before growth, (**b**–**d**) AFM images of Ge QDs on micropillars with the site-controlled pits of 80 nm in diameter after the Ge depositions of 0.77, 0.86 and 0.88 nm at 580 °C, respectively, (**e**) the dependence of the mean width (W) and height (H) of Ge QDs in pits and at periphery on the amount of Ge deposition. The scale of the color bar in AFM image is 150 nm. The black and white arrows in (**c**) indicate Ge QDs in pits and at periphery on micropillar’s top.

Figure 2a shows the typical surface morphology of a Si micropillar with deterministic pits before growth. Figure 2b–d exhibit the morphology evolution of QDs with the nominal Ge deposition of 0.77, 0.86 and 0.88 nm at 580 °C, respectively. The pit on the micropillar’s top is 80 nm in diameter before growth. In addition, two types of Ge QDs are formed on the surface of the micropillar’s top after the Ge growth. One type of QD is small and distributed at the periphery of the micropillar’s top, denoted by a white arrow in Figure 2c. The other is large and located in the desired pit, denoted by a black arrow in Figure 2c. In addition, there is generally one QD in the pit due to the small pit’s size. The QDs at the periphery form a ‘necklace’ of QDs, which is analogous to the growth of Ge on an ordinary Si micropillar without a pit [25]. Their sizes only slightly increase with the Ge deposition, as demonstrated in Figure 2e. This is distinguished from the previous result on the micropillar without pit [25]. The main reason is the appearance of additional types of QDs in pits. With the increase in Ge deposition, each pit on the micropillar’s top tends to be filled by a single QD. Moreover, the size of QD in the pit remarkably increases with the Ge deposition, as demonstrated in Figure 2e. Such different behaviors of the evolutions of two types of QDs with the Ge deposition imply that the nucleation of QD at the periphery starts earlier, while the QDs in pits grow faster in the later stage of the Ge deposition under the present growth conditions. They also demonstrate that the pit can be used to achieve precise site-controlling of Ge QDs on a microdisk. On the other hand, the competitive growth of QD at the periphery deteriorates the actual spatial matching between QDs and the WGM in a microdisk.

**Figure 3:**
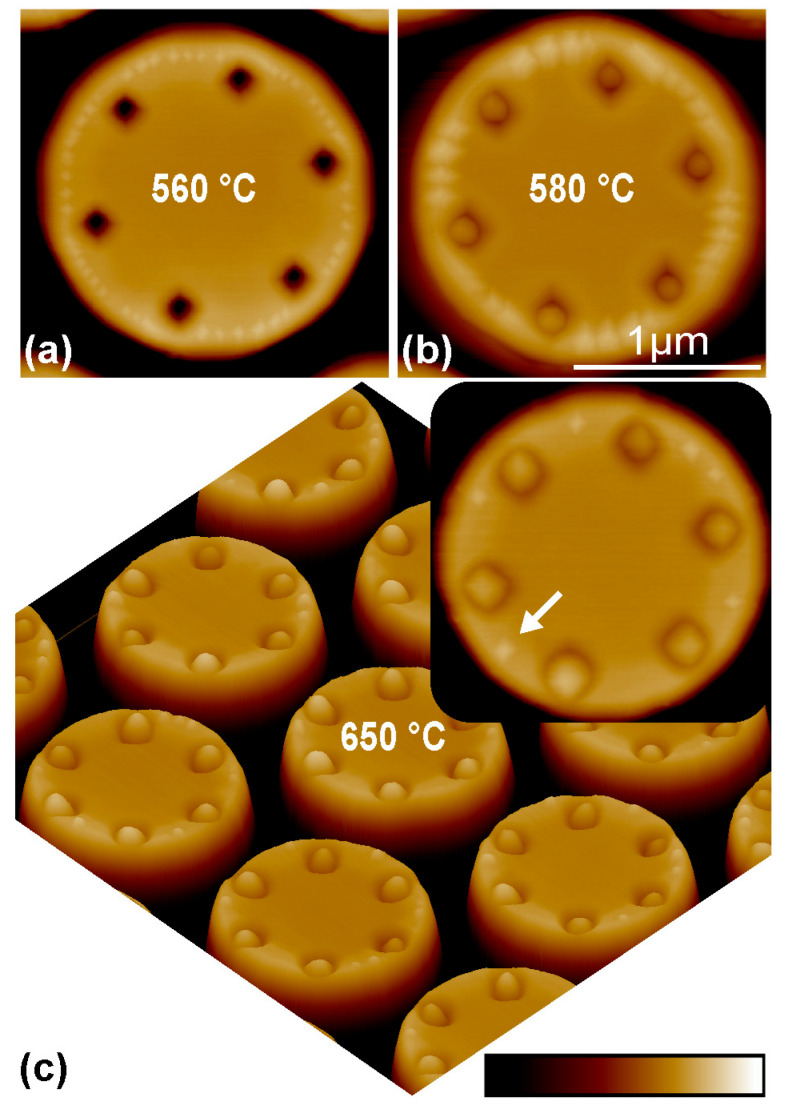
AFM images of QDs on the micropillars with pits at different temperatures. AFM images of Ge QDs on the micropillars with the site-controlled pits of 90 nm in diameter after the Ge depositions of 0.88 nm at (**a**) 560 °C, (**b**) 580 °C and (**c**) 650 °C. The white arrow in the inset of (**c**) denotes a QD at the periphery around the middle region in between the neighboring pits. The scale of the color bar is 150 nm.

To clarify the competitive growths of QD at the periphery and in pits, the effect of growth temperature is considered. Figure 3a–c show the surface morphologies after the Ge deposition of 0.88 nm on the micropillars with the pits of 90 nm in diameter at 560 °C, 580 °C and 650 °C, respectively. At 560 °C, no QD is distinguishable in pits, whereas many small QDs are distributed at the periphery of micropillar’s top. When the growth temperature of Ge is increased to 580 °C, Ge QDs appear both at the periphery and in pits on the micropillar. This is similar to the Ge growth on the micropillar with pits of 80 nm in diameter, as shown in Figure 2d. In addition, the number of QDs at the periphery decreases, and the QDs become larger with increasing temperature. These transformations are related to the increased surface diffusion length of adatoms with the growth temperature and the possible Ostwald ripening or coarsening progress [28] that describes the coalescence or redeposition of small particles into large ones. By further increasing the growth temperature to 650 °C, QDs in pits become considerably larger, whereas the QDs at the periphery tend to be smaller. Particularly, the number of QDs at the periphery is remarkably reduced, as shown in Figure 3c. Averagely, about one QD appears at the periphery in between the neighboring pits. It is generally located around the middle region in between the neighboring pits, as indicated by a white arrow in the inset of Figure 3c. Obviously, the overall volume of Ge QDs in pits is substantially increased with the growth temperature in comparison with that at the periphery. These results demonstrate that Ge adatoms are more likely to migrate into pits than to the periphery at high growth temperatures.

**Figure 4:**
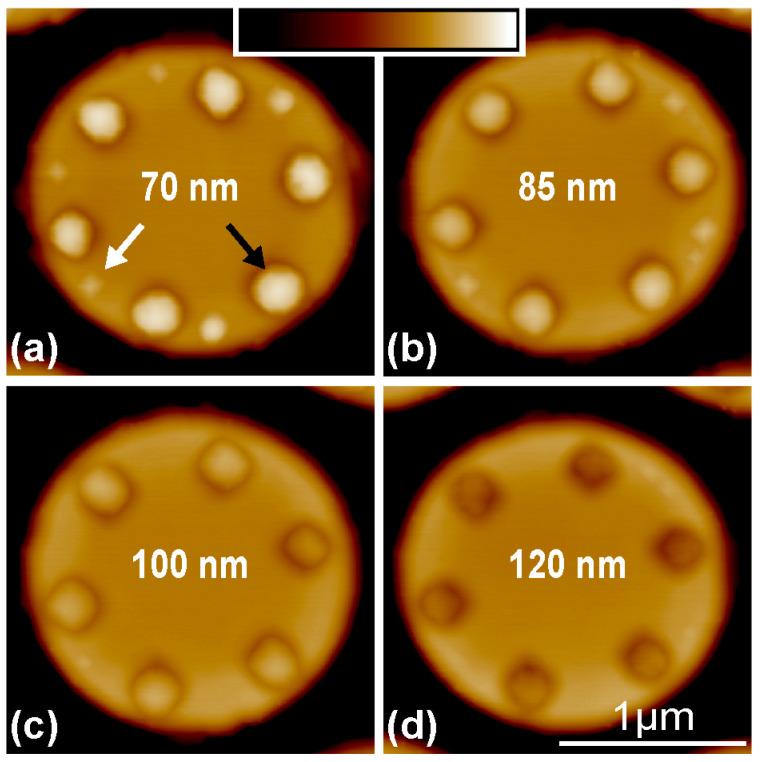
AFM images of QDs on micropillars with pits of different diameters. AFM images of Ge QDs after 0.8 nm Ge deposition at 650 °C on micropillars with the site-controlled pits of (**a**) 70 nm, (**b**) 85 nm, (**c**) 100 nm and (**d**) 120 nm in diameters. The black and white arrows in (**a**) denote Ge QD in pits and at the periphery around the middle region in between two neighboring pits. The scale of the color bar is 150 nm.

To further corroborate the preferential migration of Ge adatoms into pits at high temperatures for the precise site-controlling of Ge QDs, 0.8 nm Ge is deposited on the micropillars with pits of different diameters at 650 °C. Figure 4a–d show the AFM images of Ge QDs on the micropillars with the pits of (a) 70 nm, (b) 85 nm, (c) 100 nm and (d) 120 nm in diameter, respectively. Single Ge QD is located in each pre-patterned pit in all cases. For the pits of small diameter, e.g., less than 100 nm, additional small QDs can appear at the periphery of micropillar’s top. They are always located around the middle region in between two neighboring pits, as shown in Figure 4a,b. These results are consistent with those in Figure 3c. Moreover, the number of small QDs at the periphery tends to decrease with the increase in the pit’s diameter. For sufficiently large pits, e.g., of 100 nm or 120 nm in diameter, no QD at the periphery can be obtained. The deterministic QDs on the micropillar are actually realized, as demonstrated in Figure 4c,d.

### 3.2. The Inherent Mechanism of the Competitive Growths of QD in Pits and at Periphery of Micropillar

**Figure 5:**
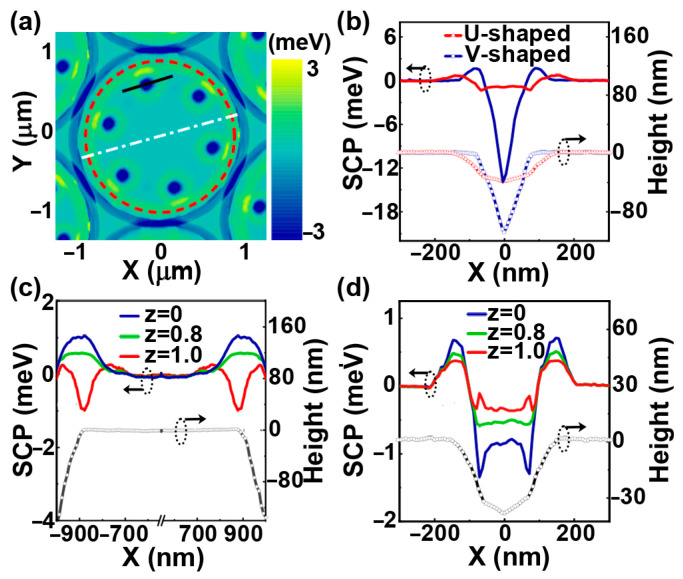
The SCP around the top of micropillar with pits. (**a**) The SCP map around the top of micropillar with pits before the Ge growth, (**b**) the distributions of SCP (line curves in the upper panel) and the height profiles (line-symbol curves in the downer panel) across different pits, (**c**) The distributions of SCP for different Z values and the height profile across the micropillar’s top (denoted by a white dashed line in (**a**)), (**d**) the distributions of SCP for different Z values and the height profile across a pit. The black line in (**a**) denotes the cross-section of pit for the blue curves in (**b**). The red dashed circle in (**a**) denotes the edge of the micropillar that is arranged in a hexagonal lattice.

It has been found that the SCP plays a significant role in the preferential growth of QDs at the periphery of microdisk [25] or micropillar’s top [29], as well as on pit-patterned substrates [30]. To disclose the mechanism of the competitive growths of QDs in pits and at the periphery, the SCP around the top of the micropillar with pits is analyzed. Generally, the SCP is given by [29,31]

(1) $$\mu =\mu_{0}+\Omega \gamma k+\Omega E_{S},$$

(2) $$E_{S}=-\frac{C}{2}\left(\frac{k}{\left|k\right|}{\left(kZ\right)}^{2}-\varepsilon^{2}\right),$$

where $\mu_{0}$ is the SCP of a flat surface, Ω is the atomic volume, γ is the surface energy per unit area, k is the surface curvature, $E_{S}$ is the local strain relaxation energy, C is an elastic constant, ε is the misfit strain, and Z is the parameter associated with the nominal thickness of the Ge film. Figure 5a shows the SCP distribution on the top of the micropillar with pits for Z = 0. The minimum SCP around the micropillar’s top appears in pits before the Ge deposition. The solid blue curve in Figure 5b shows the distribution of SCP across the pit, denoted by a black solid line in Figure 5a. It can be seen that, besides the minimum of SCP at the bottom of pit, there is a local maximum of SCP at the edge of pit. In addition, the distribution of SCP around the pit is associated with the morphologies of pit, as demonstrated in Figure 5b. Figure 5c shows the distribution of SCP across the micropillar’s top, denoted by a white dash line in Figure 5a. The SCP at the periphery of micropillar’s top also shows a local maximum. For the increase in Z with the Ge deposition, it can evolve from the local maximum to the local minimum. Such an SCP evolution is attributed to the transition of the dominance of SCP from the surface energy to the strain energy with Ge depositions [29,32]. Accordingly, beyond a critical Ge deposition, Ge adatoms tend to accumulate at the periphery of micropillar’s top and in turn facilitate the formation of Ge QDs there [32]. On the other hand, although there is a minimum of SCP at the bottom of the pit during the initial deposition, the local maximum of SCP at the edge of the pit can act as an energy barrier to suppress the migration of Ge adatoms into the pit. This barrier effect is pronounced at the low growth temperature, which delays the formation of Ge QDs in the pit. With the Ge depositions, the local maximum of SCP at the edge of pit becomes smaller, as demonstrated in Figure 5d. As a result, more and more Ge adatoms can migrate into the pit with Ge depositions. The Ge QDs can then form in the pit for a sufficient Ge deposition. In addition, they can grow faster than the QDs at the periphery of micropillar’s top during the subsequent growth. These scenarios interpret the main features of the earlier formation of QDs at the periphery of micropillar’s top and the faster growth of QDs in pits at the later growth stage at the temperature of 580 °C, which are shown in Figure 2.

At a sufficiently low growth temperature, it is difficult for Ge adatoms to migrate into the pit due to the barrier effect at the pit’s edge even at the later growth stage. Therefore, Ge QDs can hardly form in the pit, as shown in Figure 3a for the growth at 560 °C. The barrier effect at the pit’s edge becomes less pronounced with the increase in growth temperature. At a sufficiently high temperature, it can even be ignored. Consequently, the Ge adatoms around the pit preferentially migrate into the pit with the minimum of SCP, as facilitates the formation of Ge QD there. Meanwhile, a depletion region for the formation of QD around the pit exists. Accordingly, the QDs at the periphery of micropillar’s top essentially appear around the middle region in between the neighboring pits for sufficient Ge deposition, as shown in Figure 3c and Figure 4a,b for the growth at 650 °C. A larger pit is generally accompanied by a larger depletion region, which can even cover all regions in between the neighboring pits. Therefore, no QD at the periphery of micropillar’s top exists, and the QDs exclusively grow in the deterministic pits, as shown in Figure 4c,d for the growth at 650 °C on the top of micropillar with the large pits of 100 and 120 nm in diameter.

### 3.3. Prospect for Both Spatial and Spectra Matching between QDs and Cavity Modes

**Figure 6:**
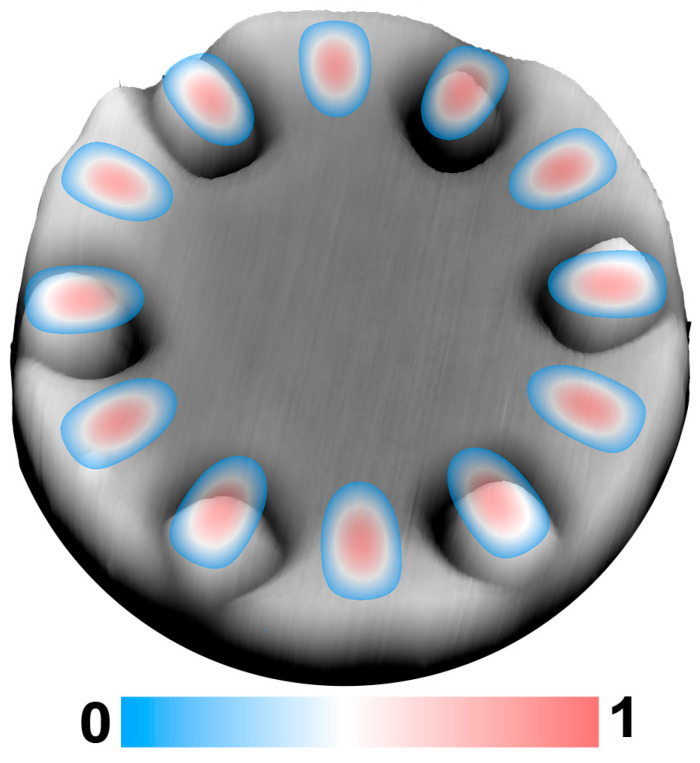
The normalized light field distribution (color image) of WGM (TE_1,6_) at the wavelength of 1.55 μm in the microdisk of 1.7 μm in diameter and the AFM image (gray image) of the micropillar of ~1.7 μm in diameter with 6 deterministic QDs after the Ge deposition of 0.8 nm at 650 °C.

The preferential migration of adatoms into pits at a sufficiently high growth temperature enables the deterministic growth of QD in any desired pit on micropillar’s top by adjusting the amount of deposition. This readily results in the spatial matching between QDs and the cavity modes of the microdisk, as well as their spectra matching. The color image in Figure 6 displays the light field distribution of WGM (TE_1,6_) at the telecommunication wavelength of 1.55 μm in a microdisk with a diameter of 1.7 μm via the FDTD method. The wavelength of WGM may be slightly changed due to the presence of pits. It can be readily compensated by tailoring the diameter and/or the thickness of microdisk [8,13]. The gray image in Figure 6 shows the surface morphology of one micropillar of ~1.7 μm in diameter with six deterministic QDs on top, which is similar to Figure 4c. The light field of WGM (TE_1,6_) contains 12 antinodes, which are located approximately at 250 nm from the microdisk’s edge. The QDs are precisely located at the next nearest neighboring antinodes of WGM (TE_1,6_), facilitating the perfect spatial matching between QDs and the cavity mode. Such an arrangement of QDs is favorable for the stronger emissions of QDs in the microdisk, given the interference of the in-phase emissions from Ge QDs coupled into the cavity mode [26]. It is worth mentioning that the site-controlled QDs in different configurations can be achieved on the top of micropillars with different arrangements of pits, whereas the growth conditions and the amount of deposition should be adjusted with the configurations of pits on the micropillar’s top. In addition, the distance between the nearest neighboring pits should be generally larger than 100 nm. To realize actual SiGe microdisks with deterministic Ge QDs, the SiGe cap layer of desired thickness should be grown, and finally, partial Si of the micropillar is selectively etched in a KOH solution [26,33], as schematically shown in steps V and VI in Figure 1. The radial position of QD in the microdisk may be slightly changed during the growth of cap layer and the chemical etching. But it can be readily compensated by adjusting the position of pit during the EBL process. For the sophisticated EBL, the uncertainty of the QD position in a microdisk can be around 10 nm. The spectra matching has generally been realized by tailoring the size (the diameter and the thickness) of microdisk that determines the wavelength of cavity mode [34] and adjusting growth conditions for the desired composition and size of QD that dominate the emission wavelength of QD [25].

Based on the above discussions, the competitive growths of QD in the pit and at the periphery of micropillars are universal for the heteroepitaxy with a lattice mismatch on semiconductor micropillars with pits, as well as microdisks with pits. The exclusive growth of QD in the pit can also be realized on other semiconductor micropillars or microdisks with desired pits by optimizing growth conditions, e.g., increasing growth temperature and/or reducing growth rate with the proper amount of deposition for large microdisks. Accordingly, the present strategy to the achievement of both spatial and spectra matching between QDs and the cavity modes in a microdisk is adaptable for other semiconductor QD/microdisk systems.

## 4. Conclusions

In summary, Ge QD can grow in the pit and/or at the periphery of Si/SiGe micropillar with predetermined pits, depending on the amount of Ge deposition and the growth temperature. The deterministic QDs with the precise locations on the micropillar’s top can be realized via the exclusive growth of QD in the pit under proper growth conditions. These unique features of heteroepitaxial growth on the top of the micropillar with pits are explained in terms of the nonuniform SCP around the micropillar’s top and the growth kinetics. Our results open a promising door to the fabrication of QD/microdisk with both spatial and spectra matching between QDs and the cavity modes of microdisk, which has always pursued the fundamental studies on CQED and the applications of innovative devices based on the QD/microdisk.

## Data Availability

The data that support the findings of this study are available from the corresponding author upon reasonable request.

## References

[1] Reithmaier J.P., Sęk G., Löffler A., Hofmann C., Kuhn S., Reitzenstein S., Keldysh L., Kulakovskii V., Reinecke T., Forchel A. (2004). Strong coupling in a single quantum dot–semiconductor microcavity system. Nature.

[2] Peter E., Senellart P., Martrou D., Lemaitre A., Hours J., Gerard J.M., Bloch J. (2005). Exciton-photon strong-coupling regime for a single quantum dot embedded in a microcavity. Phys. Rev. Lett..

[3] Srinivasan K., Painter O. (2007). Linear and nonlinear optical spectroscopy of a strongly coupled microdisk-quantum dot system. Nature.

[4] Ghosh S., Wang W.H., Mendoza F.M., Myers R.C., Li X., Samarth N., Gossard A.C., Awschalom D.D. (2006). Enhancement of spin coherence using Q-factor engineering in semiconductor microdisc lasers. Nat. Mater..

[5] Berger C., Huttner U., Mootz M., Kira M., Koch S.W., Tempel J.S., Assmann M., Bayer M., Mintairov A.M., Merz J.L. (2014). Quantum-memory effects in the emission of quantum-dot microcavities. Phys. Rev. Lett..

[6] Michler P., Kiraz A., Becher C., Schoenfeld W., Petroff P., Zhang L., Hu E., Imamoglu A. (2000). A quantum dot single-photon turnstile device. Science.

[7] Xie Z.G., Gotzinger S., Fang W., Cao H., Solomon G.S. (2007). Influence of a single quantum dot state on the characteristics of a microdisk laser. Phys. Rev. Lett..

[8] Zhou T., Tang M., Xiang G., Fang X., Liu X., Xiang B., Hark S., Martin M., Touraton M.-L., Baron T. (2019). Ultra-low threshold InAs/GaAs quantum dot microdisk lasers on planar on-axis Si (001) substrates. Optica.

[9] Wong W.W., Jagadish C., Tan H.H. (2022). III–V Semiconductor Whispering-Gallery Mode Micro-Cavity Lasers: Advances and Prospects. IEEE J. Quantum Electron..

[10] Rickman A. (2014). The commercialization of silicon photonics. Nat. Photonics.

[11] Hill M.T., Dorren H.J., De Vries T., Leijtens X.J., Den Besten J.H., Smalbrugge B., Oei Y.-S., Binsma H., Khoe G.-D., Smit M.K. (2004). A fast low-power optical memory based on coupled micro-ring lasers. Nature.

[12] He L., Ozdemir S.K., Zhu J., Kim W., Yang L. (2011). Detecting single viruses and nanoparticles using whispering gallery microlasers. Nat. Nanotechnol..

[13] Mintairov A.M., Chu Y., He Y., Blokhin S., Nadtochy A., Maximov M., Tokranov V., Oktyabrsky S., Merz J.L. (2008). High-spatial-resolution near-field photoluminescence and imaging of whispering-gallery modes in semiconductor microdisks with embedded quantum dots. Phys. Rev. B.

[14] Shih M., Hsu K., Kunag W., Yang Y., Wang Y., Tsai S., Liu Y., Chang Z., Wu M. (2009). Compact optical curvature sensor with a flexible microdisk laser on a polymer substrate. Opt. Lett..

[15] Lu Q., Chen X., Fu L., Xie S., Wu X. (2019). On-Chip Real-Time Chemical Sensors Based on Water-Immersion-Objective Pumped Whispering-Gallery-Mode Microdisk Laser. Nanomaterials.

[16] Dousse A., Lanco L., Suffczyński J., Semenova E., Miard A., Lemaître A., Sagnes I., Roblin C., Bloch J., Senellart P. (2008). Controlled Light-Matter Coupling for a Single Quantum Dot Embedded in a Pillar Microcavity Using Far-Field Optical Lithography. Phys. Rev. Lett..

[17] Fetisova M.V., Kornev A.A., Bukatin A.S., Filatov N.A., Eliseev I.E., Kryzhanovskaya N.V., Reduto I.V., Moiseev E.I., Maximov M.V., Zhukov A.E. (2019). The Use of Microdisk Lasers Based on InAs/InGaAs Quantum Dots in Biodetection. Technol. Phys. Lett..

[18] Badolato A., Hennessy K., Atature M., Dreiser J., Hu E., Petroff P.M., Imamoglu A. (2005). Deterministic coupling of single quantum dots to single nanocavity modes. Science.

[19] Sapienza L., Davanco M., Badolato A., Srinivasan K. (2015). Nanoscale optical positioning of single quantum dots for bright and pure single-photon emission. Nat. Commun..

[20] Hennessy K., Badolato A., Winger M., Gerace D., Atature M., Gulde S., Falt S., Hu E.L., Imamoglu A. (2007). Quantum nature of a strongly coupled single quantum dot-cavity system. Nature.

[21] Zhong Z., Bauer G. (2004). Site-controlled and size-homogeneous Ge islands on prepatterned Si (001) substrates. Appl. Phys. Lett..

[22] Grützmacher D., Fromherz T., Dais C., Stangl J., Müller E., Ekinci Y., Solak H.H., Sigg H., Lechner R.T., Wintersberger E. (2007). Three-dimensional Si/Ge quantum dot crystals. Nano Lett..

[23] Grydlik M., Langer G., Fromherz T., Schaffler F., Brehm M. (2013). Recipes for the fabrication of strictly ordered Ge islands on pit-patterned Si(001) substrates. Nanotechnology.

[24] Xie Z.G., Solomon G.S. (2005). Spatial ordering of quantum dots in microdisks. Appl. Phys. Lett..

[25] Wang S., Zhang N., Chen P., Wang L., Yang X., Jiang Z., Zhong Z. (2018). Toward precise site-controlling of self-assembled Ge quantum dots on Si microdisks. Nanotechnology.

[26] Zhang N., Chen P., Yan J., Peng K., Wang L., Hu H., Jiang Z., Zhong Z. (2022). Sensitively Site-Dependent Enhancement of Emissions from Ge Quantum Dots in SiGe Microdisks. Adv. Photonics Res..

[27] Kern W. (1990). The evolution of silicon wafer cleaning technology. J. Electrochem. Soc..

[28] Medeiros-Ribeiro G., Bratkovski A.M., Kamins T.I., Ohlberg D.A., Williams R.S.J.S. (1998). Shape transition of germanium nanocrystals on a silicon (001) surface from pyramids to domes. Science.

[29] Zhou T., Zeng C., Ma Q., Ma Y., Fan Y., Jiang Z., Xia J., Zhong Z. (2014). Controlled formation of GeSi nanostructures on periodic Si (001) sub-micro pillars. Nanoscale.

[30] Ma Y., Huang S., Zeng C., Zhou T., Zhong Z., Zhou T., Fan Y., Yang X., Xia J., Jiang Z. (2014). Towards controllable growth of self-assembled SiGe single and double quantum dot nanostructures. Nanoscale.

[31] Yang B., Liu F., Lagally M.G. (2004). Local strain-mediated chemical potential control of quantum dot self-organization in heteroepitaxy. Phys. Rev. Lett..

[32] Wang S., Zhou T., Li D., Zhong Z. (2016). Evolution and Engineering of Precisely Controlled Ge Nanostructures on Scalable Array of Ordered Si Nano-pillars. Sci. Rep..

[33] Zhang N., Wang S., Chen P., Zhang L., Peng K., Jiang Z., Zhong Z. (2019). An array of SiGe nanodisks with Ge quantum dots on bulk Si substrates demonstrating a unique light-matter interaction associated with dual coupling. Nanoscale.

[34] Yang S., Wang Y., Sun H. (2015). Advances and Prospects for Whispering Gallery Mode Microcavities. Adv. Opt. Mater..

